# Hydrogen Sulfide Antagonizes Chronic Restraint Stress-Induced Depressive-Like Behaviors via Upregulation of Adiponectin

**DOI:** 10.3389/fpsyt.2018.00399

**Published:** 2018-08-31

**Authors:** Qing Tian, Lei Chen, Bang Luo, Ai-Ping Wang, Wei Zou, Yong You, Ping Zhang, Xiao-Qing Tang

**Affiliations:** ^1^Institute of Neuroscience, Medical College, University of South China, Hengyang, China; ^2^Institute of Clinical Research, Affiliated Nanhua Hospital, University of South China, Hengyang, China; ^3^Institute of Clinical Research, the First Affiliated Hospital, University of South China, Hengyang, China

**Keywords:** adiponectin, chronic restraint stress, depressive-like behavior, hydrogen sulfide, synapse formation, autophagy

## Abstract

**Backgroud:** Chronic restraint stress (CRS) induces depressive-like behaviors in rodents, which involves dysregulation of hippocampal synapse formation and excessive autophagy. Adiponectin has antidepressant activity. Hydrogen sulfide (H_2_S) is a novel gasotransmitter. The present work was to investigate whether H_2_S antagonizes CRS-induced depressive-like behaviors in rats and to explore whether its potential mechanism involves ameliorated synaptic and autophagic dysregulation by upregulation of adiponectin.

**Methods:** Depressive-like behavior was analyzed by the tail suspension test (TST), novelty suppressed feeding test (NSFT), and open field test (OFT). The structure of autophagy was observed under transmission electron microscopy. The expressions of adiponectin, beclin1, and sequestosome 1 (p62/SQSTMI) protein in hippocampus were measured by Western blot. The levels of synapsin1 (SYN1) in the hippocampus were calculated by Western blot and immunofluorescence technique.

**Results:** The behavior experiments, including TST, NSFT, and OFT, showed that NaHS (a donor of H_2_S) reduced CRS-induced depressive-like behaviors. NaHS decreased the loss of hippocampal synapse as evidenced by increased the level of SYN1 in the hippocampus of CRS-exposed rats. NaHS rescued CRS-induced excessive hippocampal autophagy as evidenced by declines in the number of autophagosomes and the expression of beclin1 as well as increase in the expression of P62 in the hippocampus of CRS-exposed rats. NaHS upregulated hippocampal adiponectin expression in the CRS-exposed rats. Furthermore, neutralizing adiponectin by Anti-acrp30 reversed the protective response of NaHS to CRS-produced depressive-like behaviors as well as hippocampal synaptic disruption and excessive autophagy.

**Conclusion:** H_2_S mitigates CRS-induced depressive behavior via upregulation of adiponectin, which in turn results in amelioration in hippocampal synapse formation dysfunction and excessive autophagy.

## Introduction

Depression is a kind of serious mental disease that was caused by a variety of reasons. Chronic stress is considered as a triggering factor in the pathogenesis of depression (1, 2). It has been reported that chronic restraint stress (CRS) leads to neurological changes and depressive-like behaviors of rodents (3), which is widely applied in animal experiments to study the etiology and pathophysiology of depression (4–6). Hydrogen sulfide (H_2_S) is recognized as a neuroprotectant, which plays important roles in the central nervous system (CNS) (7). Our team verified that administration of H_2_S antagonizes depressive-like behaviors in streptozotocin-exerted diabetic rats (8). In this study, we will further explore whether H_2_S has bioavailability to prevent CRS-induced depressive-like behaviors.

Hippocampus, a critical limbic structure, is associated with mood. It has been confirmed that dysregulation of synapses and excessive autophagy in hippocampus play important roles in major depression disorder (9, 10), and that inhibition in synaptic deficits and excessive autophagy in hippocampus contributes to the antidepressant-like role of ketamine (11, 12). Thus, the present work explored whether this antidepressant-like role of H_2_S is involved in regulating hippocampal synapse formation and autophagy.

Adiponectin, as a member of adipokine family, is predominately secreted from adipose tissue (13, 14). Remarkably, adiponectin is detected in human brain and its receptors widely distributes in brain, like hippocampus and cortex (15). It has been reported that adiponectin stimulates hippocampal neurogenesis and neural stem proliferation (16, 17). Adiponectin is also associated with CNS pathologies (18). Accumulative studies reported that adiponectin is decreased in chronic social defeat stress-induced depression (19, 20). In addition, Liu et al. demonstrated that intracranial injection of adiponectin is able to ameliorate depressive-like behaviors in high fat diet-treated mice (19). Furthermore, growing evidences demonstrated that adiponectin regulates synaptic activity and autophagy (21, 22). Therefore, our hypothesis is that hippocampal adiponectin mediates the protective response of H_2_S to CRS-induced synaptic dysfunction and excessive autophagy in the hippocampus as well as depressive symptoms.

Our present work reported that H_2_S rescued CRS-induced depressive-like behaviors by elevation of hippocampal adiponectin and that suppression in hippocampal synapse loss and autophagy participates in adiponectin-mediated antidepressant efficacy of H_2_S in CRS-exposed rats.

## Materials and methods

### Reagents

Sodium hydrosulfide (NaHS, a donor of H_2_S) was obtained from Sigma (Sigma, USA). Acrp30-antibody was purchased from Santa Cruz Biotechnology (California, USA). The primary antibodies of synapsin1, beclin1, and p62/SQSTMI were purchased from Cell Signaling Technology. Bicinchoninic Acid (BCA) Protein Assay Kit was purchased from Beyotime Institute of Biotechnology in Shanghai.

### Animals

Animals that were used for all experiments are adult male Sprague-Dawley (SD) rats weighted 200–220 g (Hunan SJA Laboratory Animal Company, Changsha, Hunan, China). They were housed individually in a standard 12 h light/dark cycle and have free approach for food and water. In order to mitigate tense and anxiety of rats and eliminate influences of environmental factors, all rats were allowed to acclimatize themselves to new environment for 7 days before the experiment. This study was carried out in accordance with the recommendations of National Institutes of Health Guide for the Care and use of Laboratory Animals and was approved by the Animal Use and Protection Committee of the University of South China.

### Drugs treatments and study design

**Figure 1:**
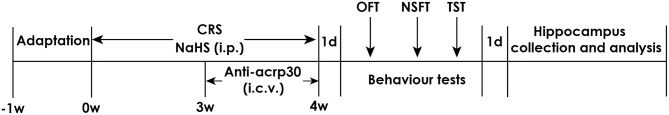
Schematic diagram of the experimental procedures. CRS, chronic restraint stress; TST, tail suspension test; NSFT, novelty suppressed feeding test; OFT, open field test; i.p., intraperitoneal injection; i.c.v., intracerebroventricular injection.

Respectively measured 1.68 mg or 5.6 mg of NaHS was diluted in phosphate-buffered saline (PBS) to equal concentration of NaHS 30 or 100 μmol/mL. Rats were subjected to CRS as well as injected with 30 or 100 μmol/kg NaHS for 4 weeks. In the fourth week, rats were treated with Anti-acrp30 (1 μg, i.c.v.) at the same time. All behavior tests were performed after 24 h of last injection. There is an interval for 2 days between each behavior test. After all tests were conducted, rats were allowed to have a rest for 1 day. After that, all rats were killed and hippocampus were collected rapidly which then stored at −80°C (Figure 1).

### Stereotaxic injection

After anesthetized with 1% sodium pentobarbital (40 mg/kg) delivered through intraperitoneal injection, rats were fixed on a stereotaxic apparatus for operation. The area central on the incision was trimmed and a aseptically cannula was implanted into lateral ventricle by reference to the following coordinates: AP: 1.0 mm, R or L:1.5 mm. For purpose of avoiding drug reflux along the injection track, the needle was drew back halfway and maintained in position for an extra 2 min ahead of being pulled out. After surgery, all rats were injected with penicillin for 3 consecutive days to prevent them from being infected.

### Chronic restraint stress (CRS) model

Chronic restraint stress was conducted as previously described (3). Rats were placed in a 50 ml stainless steel pipe for 6 h from 9 a.m. to 15 p.m. This stress was continuous for 28 days.

### Tail suspension test (TST)

The TST was carried out by reference to which described in a detailed reference (23). Briefly, each subject was tied by taping its tail closely 2 cm from the tip and then suspended in a position of 50 cm above the floor on a hook. Rats were regarded as immobile when they abandoned struggling and remained completely motionless. The time that rats kept immobility during the 6-min test was recorded.

### Novelty suppressed feeding test (NSFT)

In light of previously described (24), rats were fasting for 24 h before the test. When the experiment start, rats were placed in a test chamber (50 × 50 × 40 cm) covered with a layer of 2 cm thick wood chips from any of four corners. Food pellets were weighted and then were placed in the center of test chamber. The time from rats entering into chamber to pick up food with forelimb was defined as latency, which is a key index to reflect the degree of depression. To eliminate the influence of differences in rat appetite on the latency to feed, the rat was reintroduced to their home cage and total food intake in 10 min was recorded.

### Open field test (OFT)

The open field test (OFT) is applied to analysis the potential impacts of drug used in whole experiments on spontaneous activity. As previously described (25), rats were put in an open test chamber (60 × 60 × 40 cm) with black bottom and walls. Moreover, it is essential to ensure that the chamber was under nature light of brightness uniformity. The rat was placed into the center of test chamber and allowed to explore freely after 2 min adaption. The total distance in 5 min was recorded. Meanwhile, the chamber was cleansed by ethyl alcohol to eliminate effect of smell on spontaneous activity of next subject.

### Transmission electron microscopy (TEM)

The collected hippocampus tissues were sliced into 1 mm^3^ blocks, and were immediately placed in 2% glutaraldehyde overnight for fixation at 4°C. After immersed in 1% osmium tetroxide for 2 h, the blocks were dehydrated using graded ethanol and then embedded in epoxy resin. Next, the blocks were sliced into ultrathin sections (70 nm) using an ultramicrotome (Leica, Germany, EM UC6). Finally, the micrographs were captured under transmission electron microscope (JEOL, Japan, JEM1230).

### Western-blot analysis

Supernatant were collected of tissue homogenate and total protein concentration was quantified using BCA Protein Assay Kits. After mixed with 5 × loading buffer and heated at 100°C for 5 min, protein lysates were loaded on sodium dodecyl sulfate-polyacrylamide gel for electrophoresis and subsequently transferred onto PVDF membrane using wet transfer system. The blots were blocked using 5% skim milk in TBST buffer (50 mmol/L Tris-HCl, pH 7.5,150 mmol/L NaCl, 0.1% Tween-20) for 2 h and were respectively incubated with primary antibodies against adiponectin, SYN1, beclin1, and P62 (1:1,000), and β-actin (1:2,000) overnight at 4°C. Subsequently, membranes were washed using TBST for 3 × 10 min and were incubated with secondary antibody conjugated to horseradish peroxidase (1:5,000) for 2 h. Finally, protein membranes were washed in the same way and bands were visualized by enhanced chemiluminescence system (BeyoECL Plus kit, Beyotime, P0018). The β-actin was set as a loading control and the signal of immunoblot was analyzed by AlphaImage2200 software.

### Immunofluorescence technique

Paraffin-embedded sections were de-waxed and treated with EDTA contained buffer for antigen retrieval in microwave. Subsequently, sections were coated in autofluorescence eliminator for 5 min before washing. After 3% BSA was applied for 30 min to block nonspecific staining, the primary antibodies were added overnight at 4°C. Sections were washed for 3 × 5 min next day and corresponding secondary antibodies were added for 50 min at room temperature. DAPI was added to staining nuclear for 10 min. After washed in the same way, sections were covered with standard mounting media. Images were captured using microscope (Nikon, Japan, NIKON ECLIPSE C1).

### Statistical analysis

Statistical analysis of all data was conducted using SPSS 18.0 software. All values are exhibited as the mean ± SEM. One-way ANOVA as well as LSD-t are applied to calculate variance as well as to analyze multiple comparisons between groups, respectively. The standard of statistical difference was set at P < 0.05.

## Results

### H_2_S antagonizes CRS-induced depressive-like behaviors

**Figure 2:**
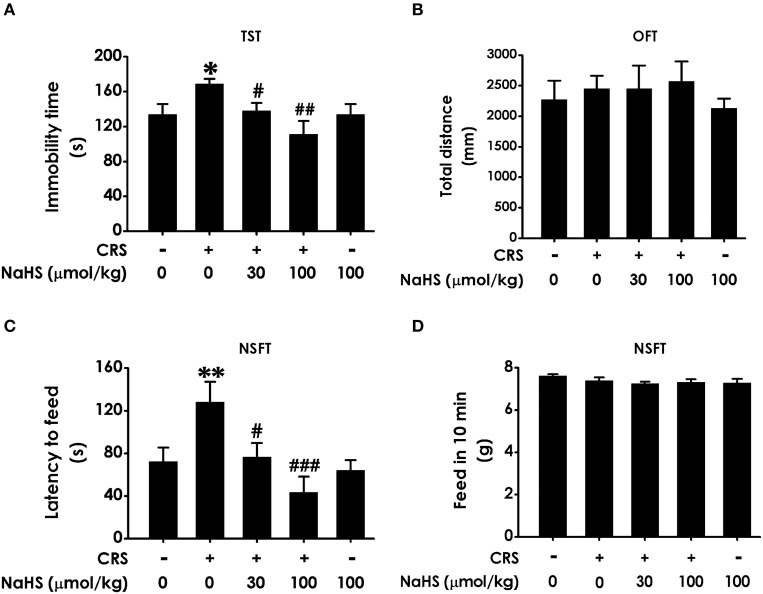
Effect of H_2_S on the depressive-like behaviors in CRS-treated rats. SD rats were cotreated with CRS and NaHS (30, 100 μmol/kg, i.p.) for 4 w. Rats were underwent TST **(A)**, OFT **(B)**, and NSFT **(C,D)** to evaluate depressive-like behaviors. Values are expressed as the means ± SEM (*n* = 6–10). *P < 0.05, **P < 0.01, vs. control group; ^#^*P* < 0.05, ^##^*P* < 0.01, ^###^*P* < 0.001, vs. CRS-treated alone group.

In TST, treatment with NaHS markedly shorten the immobility time of CRS-exposed rats and NaHS (100 μmol/kg) treated alone didn't affect the immobility time of normal rats (Figure 2A), indicating the antidepressive-like action of H_2_S. To eliminate the nonspecific motoric effects of NaHS and CRS on behaviors in the TST, the spontaneous activity of rats were assessed. As shown in Figure 2B, treatment with NaHS or STZ alone didn't have any effect on total distance in normal rats, suggesting that alteration in immobility time in TST was not due to the difference in spontaneous activity. In NSFT, the latency to feed in CRS-exposed rats was obviously decreased after administration of NaHS, while NaHS alone didn't change it in normal rats (Figure 2C). In this task, the feed in 10 min of each group in their home cage had no significant difference (Figure 2D), which excluded the possibility that alteration in the latency to feed was due to the difference in appetite and feeding. These data demonstrated that H_2_S alleviates CRS-induced depressive-like behaviors.

### H_2_S protects against CRS-generated synaptic dysfunction in the hippocampus

**Figure 3:**
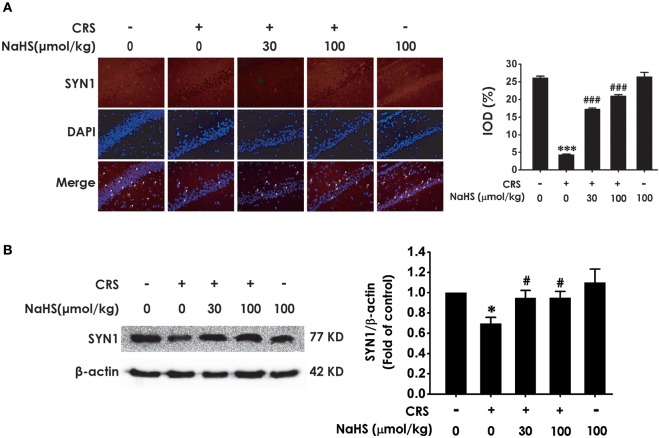
Effects of H_2_S on the suppressed synapse formation in the hippocampus of CRS-treated rats. SD rats were cotreated with CRS and NaHS (30, 100 μmol/kg, i.p.) for 4 w. The levels of hippocampal SYN1 were analyzed by immunofluorescence technique [**(A)**, magnification × 400] and western blot **(B)**. β-actin was used as an internal control. Values are expressed as means ± SEM (*n* = 3). *P < 0.05, ***P < 0.001, vs. control group; ^#^*P* < 0.05, ^###^*P* < 0.001, vs. CRS-treated alone group.

Next, we investigated the role of H_2_S in synaptic dysfunction induced by CRS in the hippocampus by measuring the expression of hippocampal SYN1. Immunofluorescence analysis exhibited NaHS (30 or 100 μmol/kg, i.p.) obviously rescued CRS-induced decline in hippocampal SYN1-positive cells of rats (Figure 3A). Western blot analysis also showed that supplement of NaHS significantly elevated the expression of SYN1 in the hippocampus of CRS-treated rats (Figure 3B). In addition, NaHS (100 μmol/kg, i.p.) alone exerted no effect on the level of hippocampal SYN1 (Figures 3A,B). These data indicated that H_2_S protects against CRS-impaired synapse formation in the hippocampus.

### H_2_S suppresses CRS-exerted excessive autophagy in the hippocampus

**Figure 4:**
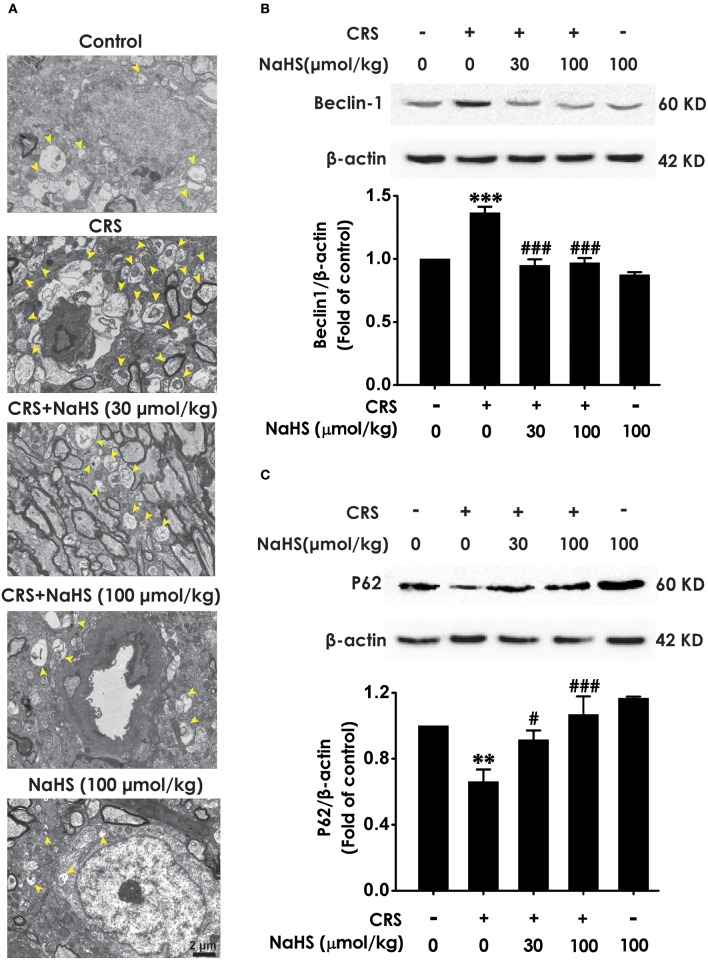
Effect of H_2_S on the excessive autophagy in the hippocampus of CRS-exposed rats. SD rats were cotreated with CRS and NaHS (30, 100 μmol/kg, i.p.) for 4 w. The number of hippocampal autophagosomes **(A)** was detected by transmission electron microscopy. The arrowhead marked autophagosomes. The expression of beclin1 **(B)** and P62 **(C)** were detected by western blot and β-actin was used as an internal control. Values are expressed as means ± SEM (*n* = 3). **P < 0.01, ***P < 0.001, vs. control group; ^#^*P* < 0.05, ^###^*P* < 0.001, vs. CRS-treated alone group.

To determine wherther the antdepressant-like role of H_2_S involves in the change of hippocampal autophagy in CRS-exposed rats, the effects of H_2_S on the number of autophagosomes and the expression of beclin1 as well as P62 in the hippocampus of CRS-exposed rats were detected. The number of autophagosomes was accumulated in the hippocampus of CRS-exposed rats, while application of NaHS (30 or 100 μmol/kg, i.p.) dramatically reduced the number of autophagosomes (Figure 4A) in the hippocampus. Rats submitted to CRS had an increase in the expression of beclin1 (Figure 4B) and a decrease in the expression of P62 (Figure 4C), while adminstration of NaHS (30 or 100 μmol/kg/d, i.p.) reversed these changes as indicated by declined expression of beclin1 and elevated expression of P62 in the hippocampus. Meanwhile, NaHS alone didn't alter these autophagic markers in normal rats, which suggested the antagonistic actin of H_2_S on CRS-exerted excessive autophagy in the hippocampus.

### H_2_S up-regulates the expression of adiponectin in the hippocampus of CRS-exposed rats

**Figure 5:**
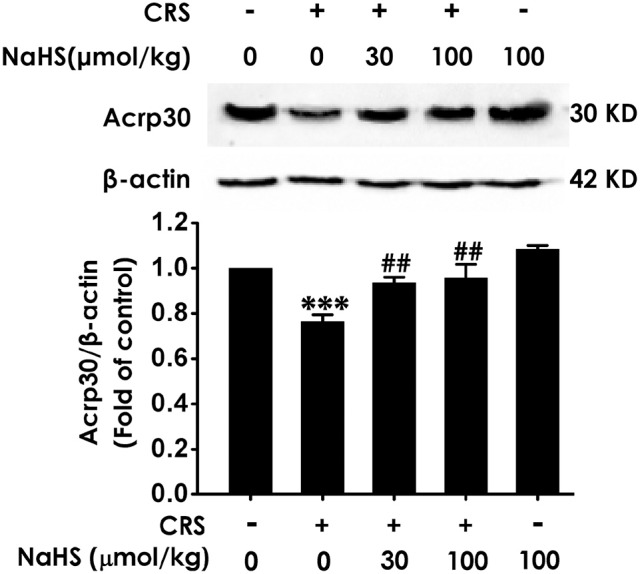
Effect of H_2_S on the expression of Adiponectin in the hippocampus of CRS-treated rats. SD rats were cotreated with CRS and NaHS (30, 100 μmol/kg, i.p.) for 4 w. The expression of hippocampal adiponectin (Acrp 30) was measured by western blotting and β-actin was used as an internal control. Values are expressed as means ± SEM (*n* = 3). ***P < 0.001, vs. control group; ^##^*P* < 0.01, vs. CRS-treated alone group.

To understand the role of adiponectin in H_2_S-elicited antidepressant-like activity, we assessed whether H_2_S affects the expression of adiponectin in the hippocampus of CRS-exposed rats. CRS-treated group showed a sharply decrease of adiponectin compared to control group. More importantly, treated with NaHS (30 or 100 μmol/kg/d, i.p.) significantly increased the expression of adiponectin in CRS-exposed rats (Figure 5). In addition, NaHS alone exerted no effect on adiponectin expression (Figure 5). Taken together, H_2_S elevated the expression of hippocampus adiponectin in the CRS-treated rats.

### Neutralizing adiponectin reverses the antidepressant response of H_2_S in CRS-exposed rats

**Figure 6:**
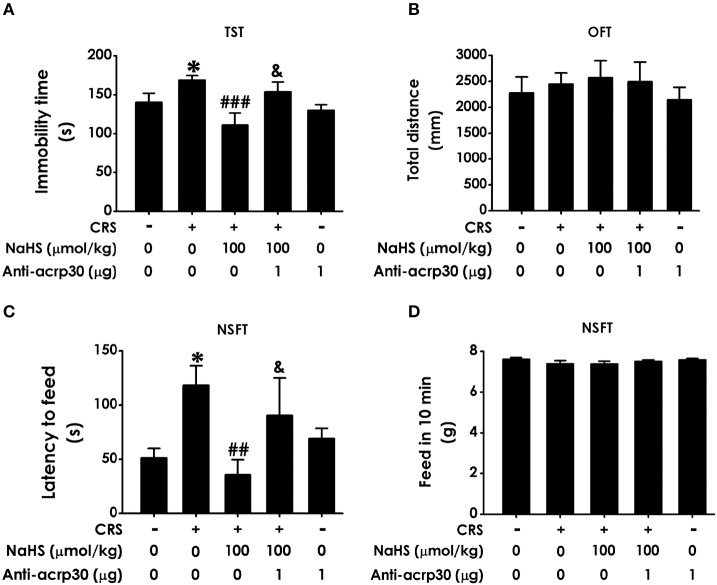
Effect of Anti-acrp30 on H_2_S-elicited antidepressant-like function in CRS-exposed rats. After cotreatment with CRS and NaHS (100 μmol/kg, i.p.) for 3 w, SD rats were further co-administrated with Anti-acrp30 (1 μg/d, icv) for 1 w. TST **(A)**, OFT **(B)**, and NSFT **(C,D)** were employed to assess depressive-like behaviors. Values are expressed as means ± SEM (*n* = 6–10). *P < 0.05, vs. control group; ^##^*P* < 0.01, ^###^*P* < 0.001, vs. CRS-treated alone group, ^&^*P* < 0.05, vs. cotreated with CRS and NaHS (100 μmol/kg, i.p.) group.

To further examine the mediatory role of adiponectin in H_2_S-elicited antidepressant-like effect, we tested whether Anti-acrp30, a neutralizing antibody of adiponectin, reverses the protective action H_2_S on CRS-induced depressive-like behaviors. As exhibited in Figure 6A, the immobility time in the CRS and NaHS-cotreated rats in TST was sharply increased by administration of Anti-acrp30 (1 μg/d, i.c.v.). The total distance in 5 min in OFT has no significance among five groups (Figure 6B), which eliminated the possible impact of Anti-acrp30 on spontaneous activity. In the NSFT, Anti-acrp30 dramatically increased the latency to feed in the cotreatment with NaHS and CRS rats (Figure 6C) and the total feed in 10 min has no significant change among all groups (Figure 6D). Taken together, these data indicated that inhibition of adiponectin reverses the antidepressant-like function of H_2_S in CRS-treated rats.

### Neutralizing adiponectin abrogates the ameliorated effect of H_2_S on CRS-induced synapse formation dysfunction

**Figure 7:**
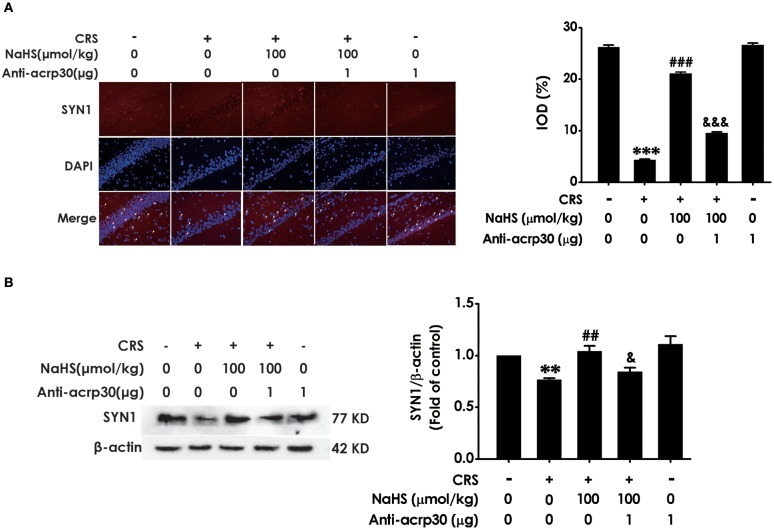
Effect of Anti-acrp30 on H_2_S-alleviated synapse formation disorder in CRS-exposed rats. After cotreatment with CRS and NaHS (100 μmol/kg, i.p.) for 3 w, SD rats were further co-administrated with Anti-acrp30 (1 μg/d, icv) for 1 w. The levels of hippocampal SYN1 were analyzed by immunofluorescence technique [**(A)**, magnification × 400] and western blot **(B)**. β-actin was used as an internal control. Values are expressed as means ± SEM (*n* = 3). **P < 0.01, ***P < 0.001, vs. control group; ^##^*P* < 0.01, ^###^*P* < 0.001, vs. CRS-treated alone group, ^&^*P* < 0.05, ^&&&^*P* < 0.001, vs. cotreated with CRS and NaHS (100 μmol/kg, i.p.) group.

For the purpose of confirming the functional requirement of adiponectin in the ameliorated role of H_2_S in CRS-elicited synaptic dysfunction, we detected the impact of Anti-acrp30 on the expression of hippocampus SYN-1 in the NaHS and CRS-cotreated rats. Immunofluorescence analysis exhibited that Anti-acrp30 (1 μg/d, i.c.v.) sharply prevented NaHS from increasing the level of SYN1-positive cells in the hippocampus of CRS-exposed rats (Figure 7A). Western blot analysis also showed that Anti-acrp30 abolished the upregulatory effect of NaHS on the hippocampus SYN1 expression of CRS-treated rats (Figure 7B). Moreover, Anti-acrp30 alone didn't change the level of SYN1. These results revealed that neutralizing adiponectin blocks the antagonistic action H_2_S in CRS-triggered disruption in hippocampal synapse formation.

### Neutralizing adiponectin reverses the inhibitory role of H_2_S in CRS-exerted excessive autophagy

**Figure 8:**
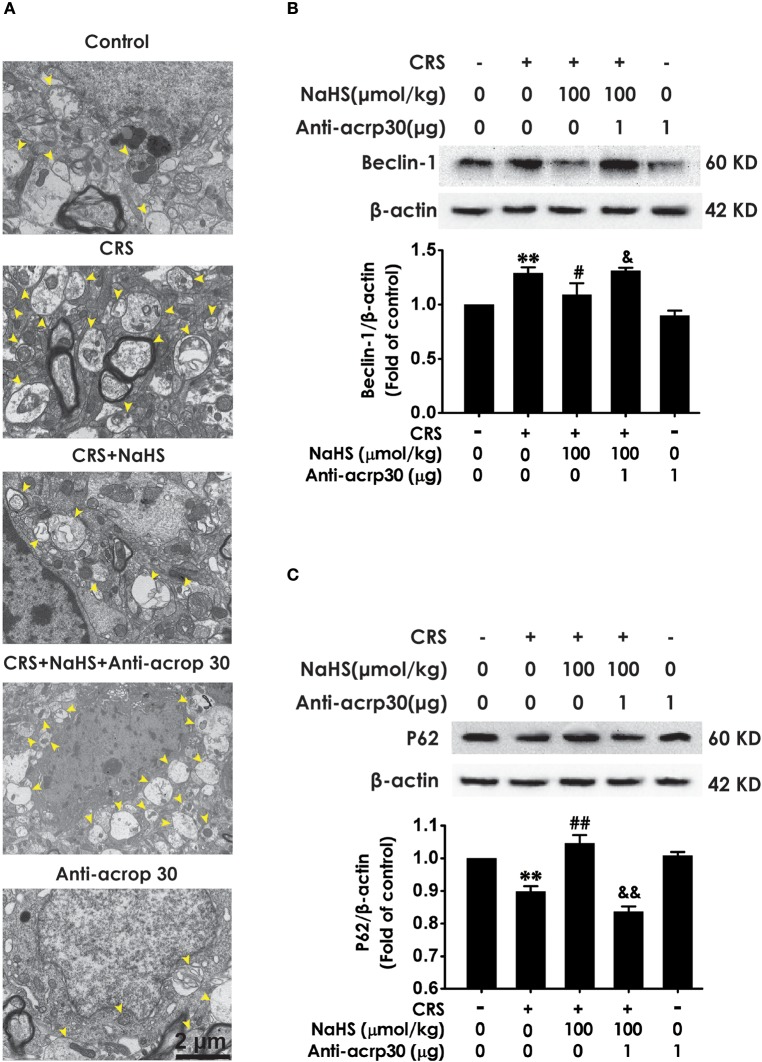
Effect of Anti-acrp30 on H_2_S-elicited suppression in CRS-induced excessive hippocampal autophagy. After cotreatment with CRS and NaHS (100 μmol/kg, i.p.) for 3 w, SD rats were further co-administrated with Anti-acrp30 (1 μg/d, icv) for 1 w. The number of hippocampal autophagosomes **(A)** was detected under transmission electron microscopy. The arrowhead marked autophagosomes. The expression of beclin1 **(B)** and P62 **(C)** in the hippocampus were detected by western blot and β-actin was used as an internal control. Values are expressed as means ± SEM (*n* = 3). **P < 0.01, vs. control group; ^#^*P* < 0.05, ^##^*P* < 0.01, vs. CRS-treated alone group, ^&^*P* < 0.05, ^&&^*P* < 0.05, vs. cotreatment with CRS and NaHS (100 μmol/kg, i.p.) group.

To identify whether adiponectin is indispensable for H_2_S-elicited suppression in the hippocampal excessive autophagy of CRS-exposed rats, we detected the impact of Anti-acrp30 on the protection of NaHS against CRS-induced excessive autophagy in the hippocampus. Anti-acrp30 dramatically prevented NaHS from increasing the number of autophagosomes (Figure 8A) and the expression of beclin1 (Figure 8B) and decreasing the expression of P62 (Figure 8C) in the hippocampus of CRS-exposed rats. Additionally, Anti-acrp30 alone exerted no effect on autophagic markers. These data suggested that neutralization of adiponectin reversed the inhibitory action of H_2_S in the excessive autophagy of hippocampus driven by CRS.

## Discussion

Chronic stress is a triggering factor in the occurrence and progression of depression, which greatly threatened human health. The present work was to explore the antidepressant role of H_2_S in CRS-induced depressive-like behaviors and the underlying mechanisms. In this study, we demonstrated that H_2_S prevented CRS-induced depression-like behaviors, hippocampal synaptic disorder and excessive autophagy as well as upregulated the expression of hippocampal adiponectin in CRS-exposed rats. Moreover, Neutralization of adiponectin reversed these protective effects of H_2_S on CRS-induced depression, disrupted synapse formation, and excessive autophagy. These data uncovered that H_2_S antagonizes CRS-induced depressive-like behaviors, involving promotion in hippocampal synapse formation and suppression in hippocampal excessive autophagy, via upregulation of hippocampal adiponectin.

Chronic restraint stress is a well-established rodent model to simulate depression-like syndrome (3, 26). Hippocampus, which plays important role in depression, is more susceptible to CRS damage (27). H_2_S is a new neuromodulator and gas signaling molecules. Several lines evidences proved that physiological concentration of H_2_S plays essential roles in central neuron system (28, 29), especially in hippocampus (30). It has reported that H_2_S can stay in the blood for more than 1 h and enter the brain through the blood-brain barrier (31). Furthermore, increased level of H_2_S in the brain was observed after intraperitoneal injection of H_2_S (32). More importantly, our previous study has proved that exogenous administration of H_2_S protects against depressive-like behaviors in streptozocin-induced diabetic rats (8). Therefore, it's necessary to investigate the beneficial role of H_2_S in CRS-induced depressive-like behaviors. Rats were subjected to CRS for consecutive 28 days and intraperitoneally injected NaHS for 4 weeks simultaneously. Subsequently, depressive-like behaviors were detected by TST, NSFT, and OFT. Consistent with previous study (3), CRS induced depressive-like behaviors. Treatment with NaHS significantly decreased the immobility time in TST of CRS-exposed rats. Meanwhile, OFT showed that there has on statistical difference of total distance among five groups, which excluded the possible influence of NaHS on the spontaneous activity. These data suggested the antidepressant-like role of H_2_S in this CRS model. In addition, administration of NaHS reduced the latency to feed in NSFT in CRS-exposed rats and the feed in 10 min has no significant difference among five groups. Taken together, H_2_S rescues depressive-like behaviors in CRS-exposed rats.

Synapsin1 (SYN1), as a synapse-associated protein, is expressed in presynaptic membrane and regulates synapse formation (33). Growing studies indicated that alteration of SYN1 is intimately associated with stress-induced depression (34, 35) and that enhancement in SYN1 participates in antidepressant process (36, 37). Autophagy is a maintaining neuronal homeostasis process that transports damaged contents to lysosomes for degradation (38). Previous research found that CRS induces elevated autophagy (39). Furthermore, mTOR signaling in depression is downregulated and this deficiency was effectively rescued by ketamine (40). Therefore, question that the effect of H_2_S on CRS-generated synaptic and autophagic dysregulation is necessary to be solved. We found that H_2_S exerted antagonistic action in CRS-induced hippocampal synaptic dysfunction indicated by the increased SYN1 level in the hippocampus of CRS-exposed rats. In addition, H_2_S attenuated CRS-induced hippocampal autophagic activation as evidenced by decreases in the number of autophagosomes and the expression of beclin1 as well as an increase in the expression of P62 in the hippocampus of CRS-exposed rats. Collectively, these data demonstrated that the protection of H_2_S against CRS-caused depressive-like behaviors involves in the ameliorations in synapse formation disorder and excessive autophagy in the hippocampus.

Adiponectin, as a key adipokine, is secreted by adipocytes. It acts an critical role in CNS (41). It has been pointed that major depression disorder patients exhibit declined adiponectin level (42). Liu et al. discovered that inhibition of adiponectin by intracerebroventricular injecting adiponectin neutralizing antibody produces depressive-like behaviors as well as enhances susceptibility to stress, and supplement of adiponectin exerts antidepressant-like effect (19). Recent studies reported that adiponectin mediates the antidepressant-like role of exercise as well as ketamine (16, 43). Our present study showed that H_2_S rescued CRS-induced the downregulation of adiponectin in the hippocampus. Further results showed that neutralized adiponectin by Anti-acrp30 reversed the antidepressant-like effect of H_2_S in CRS-exposed rats, which demonstrated that adiponectin mediates the protective role of H_2_S in CRS-exerted depressive-like behaviors. However, what caused the reduction of hippocampal adiponectin in the CRS model still remains unknown. Liu et al. reported that plasma adiponectin was decreased in chronic social defeat model (19). Even so, further research is needed to dig deeper to find out the change of adiponectin that entered into the brain. In addition, neutralizing adiponectin abrogated H_2_S-elicited facilitation in hippocampal synapse formation and suppression in hippocampal excessive autophagy in the CRS-exposed rats. These findings suggested that adiponectin-mediated protective action of H_2_S on CRS-induced depressive-like behaviors is via enhancement in hippocampal synapse formation and suppression in hippocampal excessive autophagy.

In conclusion, our work proved that H_2_S increases the level of hippocampal adiponectin as a result of promoting hippocampal synapse formation and suppressing hippocampal autophagy in CRS model, and thereby alleviates CRS-induced depression-like behaviors. Taken together, our data further confirmed the antidepressant-like role of H_2_S in CRS model and suggested that therapies directed at heightening pharmacological properties of H_2_S hold broad applicability for treating depressive disorder.

## Author contributions

X-QT and A-PW designed the study. QT, LC, and BL performed experiments. WZ, YY, and PZ conducted the analysis. QT wrote the manuscript and X-QT made necessary modifications to manuscript. All authors reviewed the manuscript.

### Conflict of interest statement

The authors declare that the research was conducted in the absence of any commercial or financial relationships that could be construed as a potential conflict of interest.
